# Influence of Dapagliflozin on Glycemic Variations in Patients with Newly Diagnosed Type 2 Diabetes Mellitus

**DOI:** 10.1155/2016/5347262

**Published:** 2016-09-21

**Authors:** Feng-fei Li, Gu Gao, Qian Li, Hong-hong Zhu, Xiao-fei Su, Jin-dan Wu, Lei Ye, Jian-hua Ma

**Affiliations:** ^1^Department of Endocrinology, Nanjing First Hospital, Nanjing Medical University, Nanjing, China; ^2^National Heart Research Institute Singapore, National Heart Centre Singapore, Singapore

## Abstract

*Objectives*. To observe changes in blood glycemic variations and oxidative stress level before and after dapagliflozin treatment in patients with newly diagnosed T2DM.* Methods*. This was a randomized, double-blind, placebo-controlled, phase 3 trial. A total of 28 patients with newly diagnosed T2DM with HbA_1c_ levels of 7.5–10.5% were randomly selected to receive dapagliflozin or placebo treatment for 24 weeks. After baseline data were collected, we analyzed glycemic variations and plasma 8-iso PGF_2*α*_ level at baseline and at the endpoint. Primary outcome was the changes of mean amplitude glycemic excursion (MAGE) within groups.* Results*. After 24-week dapagliflozin therapy, our data showed the significant improvement of MAGE with dapagliflozin therapy (*P* = 0.010). Compared with control group, patients in dapagliflozin group exhibited reduction in 24-hour MBG (*P* = 0.026) and lower mean plasma glucose concentrations, especially during periods from 2400 to 0200 and 1300 to 1800 (*P* < 0.05, resp.). In addition, plasma 8-iso PGF_2*α*_ level was notably decreased in the treatment group compared to the control group (*P* = 0.034).* Conclusions.* In conclusion, this study shows the ability of dapagliflozin to improve glycemic variations and associate with reduction of oxidative stress in patients with T2DM, which may benefit the cardiovascular system.

## 1. Introduction

Dapagliflozin, a member of sodium-glucose cotransporter-2 (SGLT2) inhibitors family, exhibits glucose-lowering effect in patients with T2DM [1, 2]. Treatment with dapagliflozin has been shown to improve glycemic control and reduce body weight and systolic blood pressure (SBP) [3–8] without increase in hypoglycemia [9]. Studies verified that the dapagliflozin is well tolerated and associated with sustained reductions in HbA_1c_, SBP, and body weight over 2–4 years in patients with T2DM [9, 10]. In older patients with longstanding T2DM, dapagliflozin is also well tolerated and achieved improvement of glycemic control without an increase of hypoglycemic episodes [11]. However, the clinical efficacy and tolerability need full elucidation in older patients with comorbidity [12]. We recently observed that dapagliflozin may confer reduced atrial natriuretic peptide levels and improved glycemic control in patients with newly diagnosed T2DM, which may benefit the cardiovascular system [13].

Dapagliflozin, reduced by hyperglycemia, body weight, and SBP, effectively addressed three cerebrovascular disease (CVD) risk factors in older patients with poorly controlled T2DM and CVD [6, 11]. Microvascular and macrovascular complications are mainly [14, 15] or partially [15, 16] dependent on hyperglycemia. The acute glucose fluctuations during postprandial periods play an important role in oxidative stress [17]. Specifically, the rapid rise in postprandial blood glucose concentration induces an overproduction of peroxynitrite and nitrotyrosine [17, 18], which had more specific triggering effect on oxidative stress [17]. Moreover, study demonstrated that hyperglycemia may induce severe alterations in ionic channels conduction properties. These channels are involved in the control of RyR2 channels, and RyR2 channels may predispose the patient to ventricular arrhythmias and sudden death [19]. So we hypothesize that dapagliflozin which benefits the cardiovascular system might partially depend on the improvement of blood variations, leading to the smoothed oxidative stress in patients with T2DM.

In this study we performed 2-time 3-day Continuous Glucose Monitoring (CGM) and measured plasma 8-iso prostaglandin F_2*α*_ (8-iso PGF_2*α*_) levels at baseline and at the endpoint of dapagliflozin therapy to observe the changes of glycemic variations and oxidative stress levels in patients with newly diagnosed T2DM compared with those treated with placebo control.

## 2. Patients and Methods

This was a randomized, double-blind, placebo-controlled, phage 3 trial. The study was performed in the Department of Endocrinology, Nanjing First Hospital, Nanjing Medical University, between July 2010 and March 2012. The study was performed as described [13]. Briefly, patients with newly diagnosed or drug-naive T2DM were recruited. After the baseline parameters were assessed, patients receiving 8 weeks of lifestyle management counseling, those who continued to experience inadequate glycemic control, as defined by HbA_1c_ levels of 7.5–10.5%, were recruited. The Interactive Voice Response System (Bristol-Myers Squibb Research and Development, Lawrenceville, NJ) will assign subjects to randomly receive one of the following blinded treatment regimens in a 1 : 1 : 1 ratio: dapagliflozin 5 mg, QD; dapagliflozin 10 mg, QD; dapagliflozin 5 mg/10 mg matching placebo, QD (distributed by Bristol-Myers Squibb, Lawrenceville, NJ), for 24 weeks, and, after 4 weeks of treatment, patients lacking glycemic control (fasting blood glucose > 11.1 mmol/L) were eligible to receive another antihyperglycemic drug, such as metformin, based on their particular symptoms. Scheduled visits will occur at weeks 1 and 24. Subjects in all treatment arms will maintain the same treatment regimen. The following exclusion criteria are applied: (1) history of diabetes insipidus; (2) severe uncontrolled hypertension (systolic blood pressure ≥ 180 mmHg and/or diastolic blood pressure ≥ 110 mmHg) and use of any renin-angiotensin system blocker; (3) replacement or chronic systemic corticosteroid treatment; (4) history or current diagnosis of significant comorbid diseases, such as cardiovascular, hepatic, and renal diseases; (5) and/or positive test for islet cell autoantibodies (such as glutamic acid decarboxylase autoantibodies, islet cell autoantibodies, or insulinoma-like antigen 2), indicating the possibility of type 1 diabetes mellitus.

Before and after 24-week dapagliflozin treatment, all patients were subjected to 2-time 3-day retrospective CGM (Medtronic Incorporated, Northridge, USA) in hospital by the specialist nurse at baseline and at the endpoint. Briefly, the CGM sensor was subcutaneously embedded at Day 0 around 16:00-17:00 PM. Subjects were instructed to keep the sensor fixed and waterproof, if CGM was going well. The study nurse inputted at least 4 calibration readings every day. At Day 4, around 16:00-17:00 PM, subjects had the sensor removed, and the CGM data were saved by the investigator, as described previously [20–22]. All patients received the same energy intake during the CGM periods. All subjects were instructed to maintain a similar level of physical activity and received meals consisting of the same nutritional value and equivalent carbohydrate intake during the study.

The 24-hour mean blood glucose (MBG), the standard deviation of the MBG, the mean amplitude of glycemic excursions (MAGE), the incremental area under curve (AUC) of blood glucose above 10.0 mmol/L, the AUC above fasting plasma glucose (FPG) concentration, and the hourly MBG were recorded and calculated, as described previously [21, 22].

The plasma 8-iso prostaglandin F_2*α*_ (8-iso PGF_2*α*_) level was measured at the baseline and the completion of the study using an enzyme immunoassay method, as we previously described (Cayman Chemical Co., Ann Arbor, MI) [22, 23].

The study was approved by the appropriate independent ethics committees and regulatory authorities and was conducted in accordance with the Declaration of Helsinki and International Conference on Harmonisation Good Clinical Practice guidelines. After the purpose and procedures of the study were fully explained, all subjects provided informed consent before enrolling in the study. The clinical protocol number was MB102055.

### 2.1. Statistical Analysis

Data were analyzed with the SPSS PASW Statistics 18 Package. Shapiro-Wilk test was used to assess the distribution of data. Normally distributed and continuous variables are presented as mean ± standard deviation (SD). The mixed ANOVA model (2 × 2) test was used to compare differences within group. An independent *t*-test was used in the comparisons between groups. Bonferroni correction was followed. *P* values were two-tailed with a significance level of 5%.

## 3. Results

A total of 28 newly diagnosed T2DM patients met inclusion criteria (18 in dapagliflozin group and 10 in placebo group) and were admitted to the study. As we reported, the demographic and baseline characteristics of study subjects were similar between placebo and dapagliflozin groups [13].

**Figure 1:**
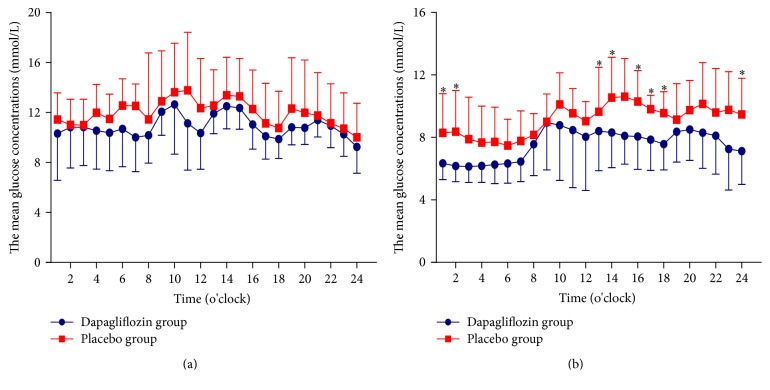
(a) The average blood glucose concentrations per hour levels in patients at baseline and (b) the average blood glucose concentrations per hour levels in patients after therapy.

There were no differences in the 24-hour MBG, the SDMBG, the MAGE, and the incremental AUC (hyperglycemia, hypoglycemia, and above FPG) and the hourly glucose blood concentrations (Figure 1(a)) within the two groups at baseline. Subjects in placebo group exhibited insignificant decreases in 24-hour MBG, the SDBG, the MAGE, and the incremental AUC of hyperglycemia after 24-week study compared with baseline, which might partially depend on the placebo effect. CGM data showed that, as expected, all patients had significant improvement of 24-hour MBG (9.12 ± 1.77 versus 7.50 ± 1.49, *P* < 0.05), SDBG (2.43 ± 1.09 versus 1.51 ± 0.42, *P* < 0.05), MAGE (5.85 ± 3.02 versus 3.48 ± 0.98, *P* < 0.05), the incremental AUC of hyperglycemia (0.69 ± 1.15 versus 0.14 ± 0.28, *P* < 0.05), and the AUC above FPG (1.68 ± 0.50 versus 1.01 ± 0.65, *P* < 0.05) after 24-week dapagliflozin treatment compared with baseline (Table 1).

Analysis comparing the two groups revealed that patients treated with dapagliflozin therapy experienced improvement of MAGE (3.48 ± 0.98 versus placebo group 5.37 ± 2.16, *P* = 0.010). Consistent with the improvement of blood glycemic excursions, a significantly decreased 24-hour MBG was exhibited in patients with dapagliflozin therapy (7.50 ± 1.49 versus 9.46 ± 1.16 mmol/L, *P* = 0.026) compared to that of control group. We also observed a numerable but insignificant reduction of the AUC (above 10 mmol/L or FPG) in patients treated with dapagliflozin compared to that of placebo group (0.14 ± 0.28 versus 0.44 ± 0.47 and 1.01 ± 0.65 versus 1.12 ± 0.80).

The average blood glucose concentrations per hour in patients in dapagliflozin group were lower than that in control group, especially from 2400 to 0200 (7.12 ± 2.13, 6.34 ± 1.04, and 6.17 ± 1.01 versus 9.47 ± 2.32, 8.29 ± 2.51, and 8.36 ± 2.64, *P* < 0.05, resp.) and 1300 to 1800 o'clock (8.40 ± 2.53, 8.31 ± 2.25, 8.10 ± 1.81, 8.06 ± 2.11, 7.85 ± 1.96, and 7.57 ± 1.65 versus 9.64 ± 2.85, 10.55 ± 2.58, 10.61 ± 2.45, 10.29 ± 1.98, 9.80 ± 0.89, and 9.55 ± 1.37, *P* < 0.05, resp.) (Figure 1(b)).

**Figure 2:**
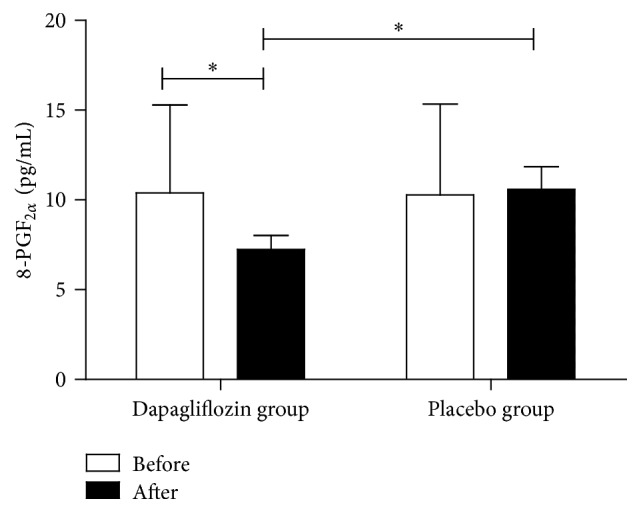
Plasma 8-PGF_2*α*_ levels in T2DM patients before (white bar) and after therapy (black bar).

To determine the effect of dapagliflozin therapy on oxidative stress in patients with newly diagnosed T2DM, we measured plasma 8-PGF_2*α*_ level, a well-recognized biomarker of oxidative stress [17]. Plasma 8-PGF_2*α*_ level was significantly decreased in the dapagliflozin group from 9.85 ± 4.91 to 7.36 ± 3.32 pg/mL (*P* = 0.022). Furthermore, plasma 8-PGF_2*α*_ level in dapagliflozin group was significantly lower than that of control group after 24-week treatment (7.36 ± 3.32 versus 10.59 ± 5.08 pg/mL, *P* = 0.034) (Figure 2).

*Safety and Tolerance*. With regard to hypoglycemia, the incremental AUC less than 3.9 mmol/L was almost the same within groups after 24-week therapy (0.02 ± 0.06 versus 0.00 ± 0.00, *P* = 0.420). One patient suffered moderate urinary tract infection during dapagliflozin therapy at 7 weeks, but then she continued the completion of study. Other patients within groups were well tolerated with dapagliflozin or placebo therapy.

## 4. Discussion

The results of the present study showed that patients treated with dapagliflozin experienced improvement of blood glycemic excursions, lowered 24-hour MBG, and insignificant reduction in AUC of >10 mmol/L and AUC above FPG. Dapagliflozin did not increase hypoglycemia. All subjects were well tolerated with the therapy.

Studies showed that the dapagliflozin is well tolerated in patients with T2DM over 2–4 years [9, 10]. In our study, 18 patients who received dapagliflozin therapy were well tolerated for 24 weeks. The increased risks of urinary tract infection and genital infection are the main dapagliflozin therapy side effects [24], and we did not observe any Serious Adverse Event (SAE), with the exception of one patient who had moderate urinary tract infection during the dapagliflozin therapy period.

As expected, our CGM data showed that patients treated with dapagliflozin were exhibiting decreased MAGE and lower 24-hour MBG, without increase in hypoglycemic episodes, compared with placebo group. The results agreed with previous studies, which demonstrated that patients treatment with dapagliflozin had benefits of improved glycemic control [3–8], with no increase in hypoglycemia [9]. SGLT-2 inhibitors lower the maximum thresholds of glucose reabsorption, thus exhibiting the glucose-lowering effect in patients with T2DM [1, 2]. Interestingly, our CGM data revealed that patients in dapagliflozin treated group had significantly reduced 24-hour MBG, especially during periods from 2400 to 0200 and 1300 to 1800, and insignificantly decreased AUC above FPG. The lower blood plasma glucose concentrations might implicate the decrease in glucose reabsorption facing hyperglycemia challenge.

Dapagliflozin reduced hyperglycemia, body weight, and SBP and effectively addressed three CVD risk factors in older patients with poorly controlled T2DM and CVD [6, 11]. However, we observed that dapagliflozin may benefit the cardiovascular system through the reduced atrial natriuretic peptide levels [13]. The smoothed blood glycemic executions might confer another protection mechanism. Acute glucose fluctuations during postprandial periods, other than chronic hyperglycemia, played a more important role in oxidative stress in patients with T2DM [17]. A rapid rise in postprandial blood glucose concentration induces an overproduction of peroxynitrite and nitrotyrosine [17, 18, 25]. Notwithstanding, continued effort has been made to suppress postprandial hyperglycemia in patients with T2DM [26]. Microvascular and macrovascular complications are mainly [14, 15] or partially [15, 16] dependent on hyperglycemia. Acute glucose fluctuations during postprandial periods played a crucial role in oxidative stress [17]. The rapid rise in postprandial blood glucose concentrations induces an overproduction of peroxynitrite and nitrotyrosine [17, 18]. Studies have shown that postprandial glucose (PPG) is an independent risk factor for cardiovascular disease [27]. By reducing postprandial excursions, oxidative and nitrosative stress can be diminished [28]. Also, chronic hyperglycemia has been demonstrated to be a risk factor for developing CVD [29, 30]. Most importantly, patients with uncontrolled blood glucose concentrations might have the modified response of cardiac resynchronization therapy (CRT) at clinical and epigenetic levels [31, 32]. Optimal glycemic control, by upregulation and differentiation of endothelial progenitor cells, may improve the myocardial salvage and affect the entity of myocardium damage extension [33]. Studies also demonstrated that diabetic patients with atherosclerotic plaques showed an altered and augmented inflammation and apoptosis response [34]. In addition, after a mean follow-up of 6.5 years, newly diagnosed T2DM patients with an increase in morning blood pressure surge had a risk of microalbuminuria, which might be induced by the fluctuation of glycemia [35]. In this study, we observed that patients with T2DM had significant improvement of blood glycemic variations after being treated with dapagliflozin for 24 weeks. Moreover, our data also showed that the oxidative stress level was significantly decreased after dapagliflozin therapy for 24 weeks. The current study indicates that there might have been a correlation between the reduction of the glycemic variability and the reduction of oxidative stress after the dapagliflozin therapy. The smoothed glycemic variations might improve the prognosis of patients with cerebrovascular disease, because glycemic fluctuations might lead to the development of atrial arrhythmias as atrial fibrillation (AF), which might be induced by the alterations in sympathetic tone regulation [36]. Indeed, AF occurred frequently in young T2DM patients and were associated with a high risk to present future cerebrovascular and silent episodes [37].

*Study Limitations*. We have limitations in the current study. In particular, the study population was relatively small, and the observation time was relatively short. In addition, more markers for oxidative stress should be employed, such as nitrotyrosine, a well-recognized biomarker to test the oxidative stress alterations and to predict clinical outcomes [38].

In conclusion, this study shows the ability of dapagliflozin to improve glycemic variations and associate with reduction of oxidative stress in patients with T2DM, which may benefit the cardiovascular system.

## Figures and Tables

**Table 1:** The blood glycemic fluctuation parameters within groups. Data were presented as means ± SD. a:  *P* < 0.05, dapagliflozin group versus placebo group.

Items	Dapa group			Placebo group			Dapa versus placebo
	Before	After	*P* value	Before	After	*P* value	*P* value (after)
MBG	10.12 ± 1.77	7.50 ± 1.49	0.031	11.01 ± 2.20	9.46 ± 1.16	0.068	0.010^a^
SDMBG	2.43 ± 1.09	1.51 ± 0.42	0.042	2.04 ± 0.62	1.75 ± 0.62	0.452	0.338
MAGE	5.85 ± 3.02	3.48 ± 0.98	0.038	5.76 ± 1.38	5.37 ± 2.16	0.357	0.026^a^
AUC > 10	0.69 ± 1.15	0.14 ± 0.28	0.140	1.73 ± 1.71	0.44 ± 0.47	0.120	0.119
AUC < 3.9	0.00 ± 0.00	0.02 ± 0.06	0.343	0.00 ± 0.00	0.00 ± 0.00	—	0.420
AUC-FPG	1.68 ± 0.50	1.01 ± 0.65	0.044	1.62 ± 0.74	1.12 ± 0.80	0.152	0.524

Dapa group: dapagliflozin group; before: before therapy; after: after therapy; MBG: mean blood glucose (mmol/L); SDMBG: standard deviation of mean blood glucose (mmol/L); MAGE: mean amplitude of glycemic excursions (mmol/L); AUC > 10: the incremental area under curve of plasma glucose > 10.0 mmol/L (mmol/L per day); AUC < 3.9: the incremental area under curve of plasma glucose < 3.9 mmol/L (mmol/L per day); AUC-FPG: the incremental area under curve above fasting plasma glucose (mmol/L per day).
